# Passive–Active Cooperative Design Method for Fall Protection in Humanoid Robot

**DOI:** 10.3390/biomimetics11090644

**Published:** 2026-09-08

**Authors:** Tian Mu, Junyao Gao, Weilong Zuo, Leilei Xie

**Affiliations:** School of Mechatronics Engineering, Beijing Institute of Technology, Beijing 100081, China; gaojunyao@bit.edu.cn (J.G.); 3220205022@bit.edu.cn (W.Z.); 3220235256@bit.edu.cn (L.X.)

**Keywords:** humanoid robot, passive–active cooperative protection, VHIP

## Abstract

Humanoid robots are highly susceptible to structural damage during irrecoverable falls due to high landing velocity, short impact duration, and high peak impact force. Inspired by human protective strategies, namely instinctive postural adjustment and soft-tissue energy absorption, this paper proposes a passive–active cooperative fall-protection method that combines pre-impact motion regulation with post-impact structural energy absorption. On the passive protection side, high-risk contact regions are identified through multi-directional fall simulations, and a multi-region, multilayer protective suit is optimized considering impact energy absorption, peak-force reduction, anti-bottoming safety, added mass, and thickness constraints. On the active protection side, a variable height inverted pendulum (VHIP) model is used to optimize the center of pressure and center of mass trajectories, reducing the terminal impact energy before ground contact. The residual impact energy is then matched with the absorption capacity of the passive protective layers, forming a unified framework that integrates pre-impact motion unloading and post-impact energy absorption. Numerical validation is performed on a MATLAB–CoppeliaSim co-simulation platform, and physical experiments are conducted on the FCR humanoid robot (approx. 50 kg, 1.65 m, 22 DOF). Compared with the unprotected case, the proposed method reduces the peak equivalent impact force from 4819.1 N to 1038.2 N, i.e., a reduction of 78.5%, demonstrating its effectiveness in attenuating impact loads and enhancing protection capability.

## 1. Introduction

Humanoid robots possess human-like morphology and exhibit strong adaptability to various environments. Therefore, they have promising application prospects in fields such as service, industrial collaboration, and disaster relief [1]. However, compared with wheeled or tracked robots, humanoid robots typically have a higher center of mass, a smaller foot support area, and more degrees of freedom. Their stability relies on accurate state estimation and low control latency, and they are highly sensitive to external disturbances [1,2,3]. Considering complex real-world working environments, such as external-force disturbances, uneven terrain, and uncertain friction conditions, robots may enter unrecoverable unstable states and fall in multiple possible directions [1,4]. Recent studies confirm that fall safety remains an active research issue: a 2025 review systematically classified humanoid fall-coping strategies into fall-state detection, fall prevention, and post-fall protection [5], while recent fall-prediction studies have used learning-based models and data-driven state analysis to increase warning lead time and identify unstable states [6,7]. In addition, DCM-based stabilization under terrain-induced time-varying disturbances and online foothold planning on uneven terrain have been developed to improve dynamic balance before a fall becomes unavoidable [8,9].

The hazards caused by robot falls are mainly in three aspects [1]. First, the impact velocity upon landing is generally high, and the kinetic energy of the falling robot is converted into contact deformation energy and internal structural energy within an extremely short period, which may cause sensor damage, and loosening of connectors. Second, local body regions may strike the ground with a very small contact area, generating excessively high local peak impact forces and stresses. Third, if the joint postures are not properly controlled during the fall, abnormal joint impacts and reverse excitation of the transmission system may occur, consequently damaging reducers, motors, and linkage mechanisms. Therefore, fall protection should not merely focus on avoiding falls, but also place emphasis on how to land safely and mitigate damage during a fall.

Existing studies on robot falling mainly focus on fall prediction, fall protection, and fall recovery [1,5]. Fall protection can be further divided into passive protection and active protection. Passive protection absorbs impact energy, prolongs collision duration, and reduces peak impact force through cushioning materials, compliant mechanisms, airbags, elastic shells, and energy-absorbing structures. Previous studies include a passive cushioning arm for forwardfall protection of humanoid robots [10], an airbag-based method for reducing fall-induced impact acceleration [11], and an active compliant impact-protection system for the hand of the WALK-MAN robot using inflatable structures [12]. Other studies have used compliant arm control, internal protection mechanisms, protective shells, and low-damage fall-sequence design to reduce fall damage [13,14,15,16,17,18]. However, most of these methods focus on specific limbs, specific landing segments, or a single falling direction, and they still lack a systematic full-body design method that simultaneously considers multiple candidate contact regions, added mass, and energy-absorption capacity [10,11,12,13,14,15,16,17,18].

Active protection relies on the robot’s sensing, planning, and control capabilities. Before or during a fall, the robot actively adjusts its CoM, support region, joint posture, and contact sequence to recover balance or reduce landing impact. Early research mainly focused on stability criteria and fall prediction, such as evaluating balance through angular-momentum variation [2], using the capture-point theory for stepping recovery after disturbance [3], and predicting falls with machine learning, energy states, and multi-sensor information [19,20,21]. Direction-changing falling control further showed that stepping and inertia shaping can alter the default falling direction and avoid sensitive targets in the environment [4]. The common goal of these methods is to identify the fall tendency before complete loss of stability and provide a trigger for subsequent protective actions.

For controlled falling, the UKEMI method proposed by Fujiwara et al. is a classical representative [22]. Inspired by the judo “breakfall” action, it actively adjusts the robot’s posture so that the robot contacts the ground with body regions that are less likely to be damaged [23]. Subsequent studies further proposed optimal falling motion planning, real-time falling motion selection, humanfall-inspired control, three-point support falling, whole-body trajectory optimization, and multi-contact planning methods [24,25,26,27,28,29]. Among them, multi-contact planning distributes one large impact into several smaller impacts by planning the ground-contact sequence of hands, knees, back, hips, and other regions, thereby reducing local damage risk [29,30]. Post-impact compliance and predictive control have also been introduced to regulate contact forces after touchdown and reduce rebound or secondary impact [31,32]. In recent years, active protection has gradually developed toward whole-body dynamics control, online optimization, learning-based strategies, and environment-assisted protection. For example, online falling control has been implemented using energy shaping and energy distribution [33]; contact-transition-tree optimization has also been used for multi-contact fall mitigation [30]. Hand-contact stabilization and wall-assisted strategies further indicate that nearby environmental supports can be exploited to arrest or redirect a fall [34,35,36]. More recent work has used whole-body dynamics with wall support to generate fall-protection trajectories for humanoid robots [37], while learning-based arm-assisted control has been explored for adaptive fall-damage reduction and recovery in legged mobile manipulators [38]. Dynamic multi-contact standing-up control has also been developed to improve post-fall recovery of humanoid robots [39]. Human-inspired fall-arrest methods based on stiffness ellipsoid optimization provide another route for distributing impact loads through appropriate upper-limb compliance [40]. Although these active protection methods can improve fall posture and contact sequence to some extent, limitations remain. First, whole-body-dynamics-based optimization must handle high-dimensional states and complex constraints, resulting in a large computational scale that can be challenging for real-time execution during fast falls [28,29,30]. Second, many methods focus mainly on pre-contact motion planning or contact-sequence regulation and do not jointly optimize structural protection and material energy absorption at the contact instant, making it difficult to balance protection effectiveness, additional weight, and motion flexibility [24,25,28,29,30,31,32,33].

To address these limitations, this paper presents a passive–active cooperative fall-protection method for humanoid robots. The basic idea is as follows: when the robot retains a certain degree of control authority, the active protection strategy uses a variable height inverted pendulum (VHIP) model to describe the dominant center of mass (CoM) dynamics during a fall [41]. A low-dimensional optimization problem is used to generate the CoP trajectory and CoM height trajectory, thereby reducing terminal impact velocity and residual impact energy. When ground contact is unavoidable, passive protection absorbs the remaining energy through a multi-region and multilayer protective suit, extends the collision duration, and reduces the peak impact force. Furthermore, the residual impact energy after active protection is coupled with the safe energy absorption capacity of the passive protective layers, thereby integrating impact reduction through motion before contact and structural energy absorption after contact within a unified safety constraint framework.

From a biomimetic perspective, human fall responses provide a natural blueprint for such a cooperative strategy. When humans face an unavoidable fall, they instinctively lower the center of mass by flexing the knees and hips and rotate the body to distribute contact loads over larger, more resilient areas such as the back and thighs. Following these innate responses, the impact energy is subsequently absorbed by soft tissues through passive deformation, which prolongs the effective impact duration and attenuates the peak forces transmitted to the skeletal structure. Thus, the active motion regulation and passive structural energy absorption in our method correspond to the two sequential mechanisms in human protective behavior, confirming the biomimetic rationale behind the proposed design.

**Figure 1:**
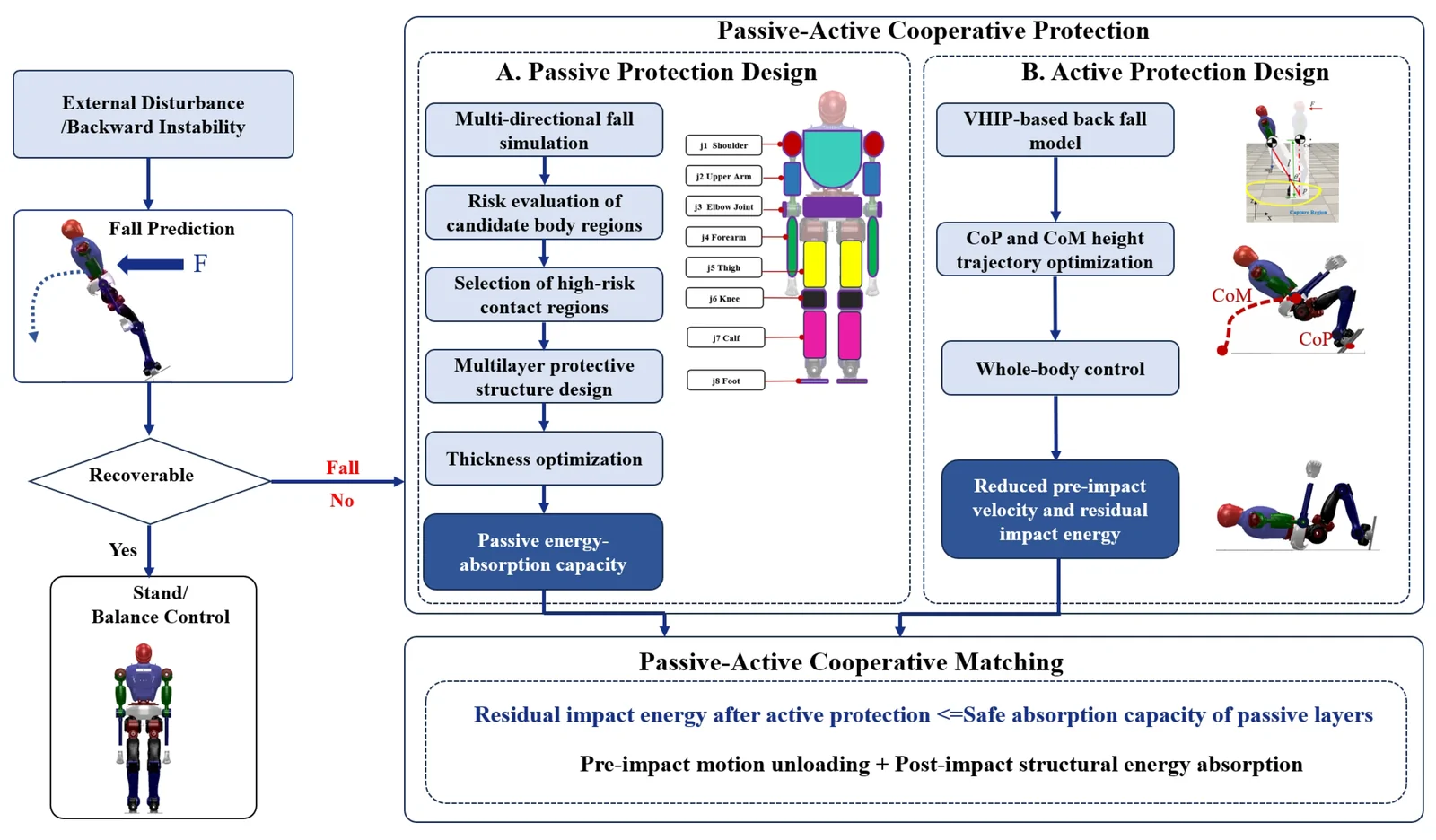
Passive–active cooperative design framework for fall protection of humanoid robot.

As shown in Figure 1, this paper constructs a passive–active cooperative design framework for fall protection of humanoid robots. The robot first performs fall prediction. If the loss-of-balance state is recoverable, it continues standing or balance control. If the fall is judged unrecoverable, active and passive protection are started. The active protection module optimizes the CoP and CoM height trajectories based on the VHIP model and generates joint-angle commands through whole body control to reduce terminal impact energy before landing. The passive protection module designs a multilayer protective structure according to the selected risk regions and uses thickness optimization to satisfy energy absorption, peak impact force, anti-bottoming, mass, and thickness constraints. By coordinating motion-based impact reduction before contact with structural energy absorption after contact, the proposed framework enables safe landing during unrecoverable falls.

The contributions of this paper are threefold.

1. A multi-region, multilayer passive protection design is proposed to identify high-risk contact regions and optimize protective-layer thicknesses under energy absorption, impact force, anti-bottoming, mass, and thickness constraints.
2. A VHIP-based active protection method is developed to optimize the CoP and CoM height trajectories, thereby reducing the pre-impact velocity and residual impact energy during a fall.
3. The proposed active–passive protection method is experimentally validated on a real humanoid robot. The results show that it effectively prevents observable damage to the robot.

## 2. Passive Protection: Thickness Optimization for a Multi-Region and Multilayer Protective Suit

To reduce impact damage when the robot contacts the ground, this paper designs a multi-region and multilayer composite protective suit according to the contact risk and impact energy of different body regions. Specifically, key protective regions are first identified by multi-directional fall simulation. Then, for each protected region, a multilayer structure composed of an outer wear-resistant layer, a middle energy-absorbing layer, and an inner cushioning layer is constructed. Finally, the layer thicknesses are used as optimization variables, and coordinated optimization between protection performance and lightweight design is achieved under constraints on energy absorption, anti-bottoming, mass, and thickness.

### 2.1. Mechanical Basis for Impact Mitigation

When an irrecoverable fall occurs, the landing damage of a humanoid robot is mainly related to the pre-impact velocity, impact duration, and energy-absorption capability of the contact region. Therefore, the proposed protection strategy is designed from both impulse and energy perspectives. For the *j*th candidate contact region under the *s*th fall scenario, let $m_{j}$ denote the equivalent impact mass, $v_{j,s}^{-}$ and $v_{j,s}^{+}$ denote the normal velocities before and after impact, respectively, and $\Delta t_{j,s}$ denote the effective impact duration. According to the impulse–momentum relationship in classical impact mechanics [42], the average normal impact force can be estimated as

(1) $${\bar{F}}_{j,s}=\frac{m_{j}\left|v_{j,s}^{-}-v_{j,s}^{+}\right|}{\Delta t_{j,s}}.$$

Since the post-impact normal velocity is usually small during ground contact, i.e., $v_{j,s}^{+}\approx 0$, the peak impact force can be approximated by introducing a correction factor $\kappa_{j}>1$:

(2) $$F_{j,s}^{\mathrm{peak}}\approx \kappa_{j}\frac{m_{j}\left|v_{j,s}^{-}\right|}{\Delta t_{j,s}}.$$

Equation (2) shows that the impact force can be reduced by decreasing the pre-impact velocity or increasing the collision duration. This explains the complementary roles of active and passive protection: active protection reduces $v_{j,s}^{-}$ before impact, while passive protection increases $\Delta t_{j,s}$ after contact.

From the energy perspective, the impact energy of the *j*th region can be approximated from the pre-impact kinetic energy [42] as

(3) $$E_{j,s}^{\mathrm{imp}}=\frac{1}{2}m_{j}{\left(v_{j,s}^{-}\right)}^{2}.$$

For an energy-absorbing protective layer, the absorbed energy corresponds to the area under its force–displacement response [43] and can be expressed as

(4) $$E_{j}^{\mathrm{abs}}=\int_{0}^{\delta_{j}^{\max}}F_{p,j}\left(\delta \right)\;d\delta ,$$

where $\delta_{j}^{\max}$ is the maximum allowable compression displacement before bottoming out. To ensure safe protection, the energy-absorption capacity of the passive layer should satisfy

(5) $$E_{j}^{\mathrm{abs}}\geq k_{E}E_{j,s}^{\mathrm{imp}},$$

where $k_{E}$ is a safety factor. This analysis provides the mechanical basis for the proposed passive–active cooperative method. Active protection reduces the impact energy before contact, and passive protection absorbs the remaining energy after contact. The two mechanisms are further coupled in the following sections through region selection, thickness optimization, and residual-energy matching.

### 2.2. Identification of Protective Regions

**Figure 2:**
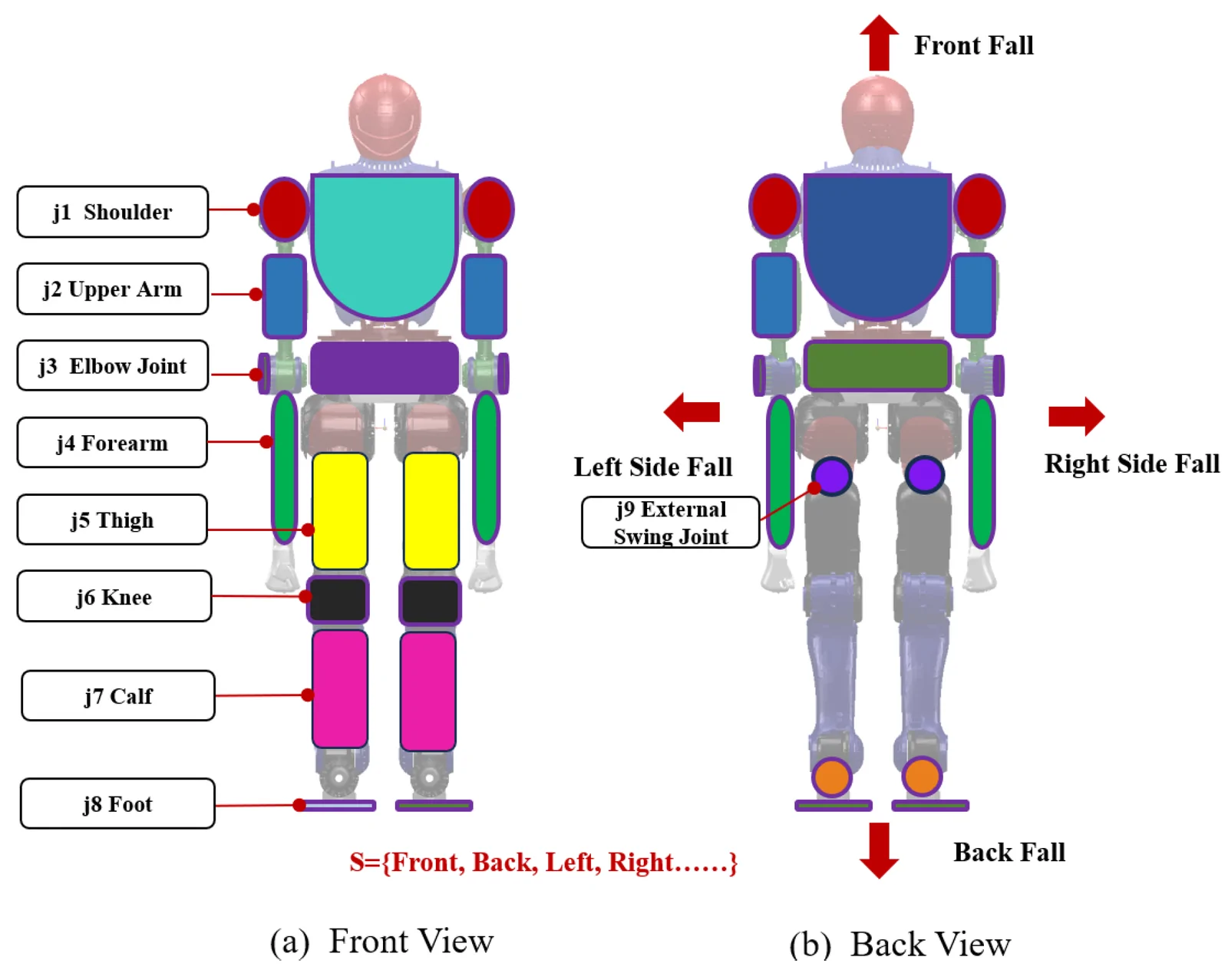
Candidate protective regions on the humanoid robot.

To determine which body regions require focused protection, the robot body is divided into *j* candidate protective regions, such as the back, hip, shoulder, elbow, knee, chest, and pelvis, as shown in Figure 2.

To determine the regions that should be protected, a combination of fall simulation verification is used. In the fall simulations, typical directions including forward, left-side, right-side, and diagonal falls are considered. The direction set is denoted by S={front,back,left,right,diagonal}. For one fall, the pre-impact energy of a robot body region is defined as [42]

(6) $$E_{0,j}^{s}\approx \frac{1}{2}m_{\mathrm{eff},j}{∥v_{j}^{s-}∥}^{2}+\frac{1}{2}{\omega_{j}^{s}}^{T}I_{\mathrm{eff},j}\omega_{j}^{s}.$$

Here, $m_{\mathrm{eff},j}$ is the equivalent impact mass of the *j*th region, $I_{\mathrm{eff},j}$ is the equivalent rotational inertia near that region, and $v_{j}^{s-}$ and $\omega_{j}^{s}$ are the linear and angular velocities immediately before contact, respectively. The maximum impact energy of the *j*th region over all falling scenarios is defined as

(7) $$E_{0,j}^{\max}=\max_{s\in S}E_{0,j}^{s}.$$

To prevent damage of the protective suit itself, a safety-margin coefficient $\kappa_{E}$ is introduced. The design impact energy used for protective-suit optimization is

(8) $$E_{0,j}=\kappa_{E}E_{0,j}^{\max},\;\kappa_{E}\geq 1.$$

The maximum peak contact force of the *j*th region over all fall scenarios is further defined as

(9) $$F_{j}^{\max}=\max_{s=1,\cdots ,N_{s}}F_{j,s}^{\mathrm{peak}}.$$

In this paper, it is estimated using the impulse relation [42]

(10) $$F_{j,s}^{\mathrm{peak}}\approx \kappa \frac{m_{\mathrm{eff},j}\left|v_{j,s}^{-}-v_{j,s}^{+}\right|}{\Delta t_{j,s}},$$

where κ is the correction coefficient from average force to peak force and $\Delta t_{j,s}$ is the equivalent impact duration.

The contact probability $P_{j}$ of the *j*th region in multiple falling scenarios is defined as

(11) $$P_{j}=\frac{1}{N_{s}}\sum_{s=1}^{N_{s}}I_{j,s},$$

where $I_{j,s}=1$ if region *j* contacts the ground in scenario *s* and $I_{j,s}=0$ otherwise. If a region is the first contact region or frequently contacts the ground in most scenarios, then $P_{j}$ is large.

Considering contact probability, impact energy, and peak impact force simultaneously, the regional risk index is defined as

(12) $$R_{j}=P_{j}\left(\beta_{E}\frac{E_{0,j}^{\max}}{E_{\mathrm{ref}}}+\beta_{F}\frac{F_{j}^{\max}}{F_{\lim,j}}+\beta_{v}\frac{v_{j}^{\max}}{v_{\mathrm{ref}}}\right),$$

with

(13) $$\beta_{E}+\beta_{F}+\beta_{v}=1,\;\beta_{E}>0,\;\beta_{F}>0,\;\beta_{v}>0.$$

Here, $E_{0,j}^{\max}$, $F_{j}^{\max}$, and $v_{j}^{\max}$ are the maximum impact energy, contact force, and pre-impact velocity of the *j*th region, respectively. $E_{\mathrm{ref}}$ and $v_{\mathrm{ref}}$ are normalization references, and $\beta_{E}$, $\beta_{F}$, and $\beta_{v}$ are weighting coefficients. $F_{\lim,j}$ is the allowable impact-force threshold determined by the structural and impact resistance of the region. A candidate region is defined as a key protective region if its risk index satisfies

(14) $$R_{j}\geq R_{\mathrm{th}}.$$

### 2.3. Objective Function of Passive Protection

To enhance robot protection, a multilayer protective structure is adopted for each selected region. Assume that each key protective region uses *L* layers of protective materials, such as an outer wear-resistant layer, a middle energy-absorbing layer, and an inner cushioning layer. Let $h_{i,j}$ denote the thickness of the *i*th layer in the *j*th region, where i=1,⋯,L,j=1,⋯,N. The total thickness of the *j*th region is $H_{j}$, where

(15) $$H_{j}=\sum_{i=1}^{L}h_{i,j}.$$

To balance protection capacity, lightweight design, and motion flexibility, the objective function is designed as

(16) $$\min_{h_{i,j}}J_{h}=\sum_{j=1}^{N}\sum_{i=1}^{L}\rho_{i}A_{j}h_{i,j}+\lambda_{\Omega }\sum_{\left(j,q\right)\in N_{b}}\sum_{i=1}^{L}{\left(h_{i,j}-h_{i,q}\right)}^{2}+\lambda_{H}\sum_{j=1}^{N}H_{j}.$$

Here, $\rho_{i}$ is the density of the *i*th material layer, $A_{j}$ is the protected area of the *j*th region, the first term represents the total mass of the protective suit, the second term is a thickness-distribution smoothness term that avoids abrupt thickness changes between adjacent regions, $\lambda_{\Omega }$ and $\lambda_{H}$ are weights, and $N_{b}$ denotes the set of adjacent body regions.

### 2.4. Constraints of Passive Protection

#### 2.4.1. Absorption Capacity Constraint

To improve the protection performance, a multilayer protective structure is adopted for each selected region. For the *i*th material layer, the energy absorbed per unit volume is calculated from the area under the compressive stress–strain curve, following the standard energy-absorption characterization of cushioning foams [43], as

(17) $$W_{i}=\int_{0}^{\alpha_{i}}\sigma_{i}\left(\varepsilon \right)\;d\varepsilon ,$$

where $\sigma_{i}\left(\varepsilon \right)$ is the compressive stress of the *i*th material at strain ε, and $\alpha_{i}$ is its allowable compressive strain. Since the onset of densification limits the useful energy-absorption stroke of cellular materials [44], $\alpha_{i}$ is defined in this study as

(18) $$\alpha_{i}=\eta_{i}\varepsilon_{d,i},\;0<\eta_{i}<1,$$

where $\varepsilon_{d,i}$ is the densification strain and $\eta_{i}$ is a safety coefficient. Assuming that each material layer covers the protected area $A_{j}$, the total energy absorbed by the multilayer structure in the *j*th region is

(19) $$E_{j}^{\mathrm{abs}}=A_{j}\sum_{i=1}^{L}W_{i}h_{i,j}.$$

Since $E_{0,j}$ is the design impact energy of the *j*th region and already includes the prescribed safety margin, the energy absorption constraint is expressed as

(20) $$E_{j}^{\mathrm{abs}}=A_{j}\sum_{i=1}^{L}W_{i}h_{i,j}\geq E_{0,j},\;j=1,\ldots ,N.$$

#### 2.4.2. Peak Impact Force Constraint

To limit the peak impact force transmitted to the robot, the force response of the protective structure is approximated by an equivalent linear relation. The absorbed energy is then expressed as

(21) $$E_{0,j}\approx \frac{1}{2}F_{p,j}^{\mathrm{peak}}\delta_{a,j},$$

where $F_{p,j}^{\mathrm{peak}}$ is the predicted peak impact force after passive protection and $\delta_{a,j}$ is the allowable compression stroke of the protective structure. The peak impact force should satisfy

(22) $$F_{p,j}^{\mathrm{peak}}\leq F_{\lim,j}.$$

For the multilayer protective structure, the allowable compression stroke is

(23) $$\delta_{a,j}=\sum_{i=1}^{L}\alpha_{i}h_{i,j}.$$

Therefore, the peak impact force constraint can be written as

(24) $$\sum_{i=1}^{L}\alpha_{i}h_{i,j}\geq \frac{2E_{0,j}}{F_{\lim,j}},\;j=1,\ldots ,N.$$

#### 2.4.3. Constraint Against Material Densification

The energy absorption performance decreases significantly when a protective material enters the densification region. Therefore, the maximum compressive strain of each layer should remain below its allowable value:

(25) $$\varepsilon_{i,j}^{\mathrm{peak}}\leq \alpha_{i},\;\alpha_{i}=\eta_{i}\varepsilon_{d,i},\;0<\eta_{i}<1.$$

Here, $\varepsilon_{i,j}^{\mathrm{peak}}$ is the maximum strain experienced by the *i*th layer in the *j*th region, $\varepsilon_{d,i}$ is the densification strain of the *i*th material, and $\eta_{i}$ is a safety coefficient. In terms of compression displacement, this constraint becomes

(26) $$\delta_{i,j}^{\mathrm{peak}}\leq \alpha_{i}h_{i,j},\;i=1,\ldots ,L,\;j=1,\ldots ,N.$$

#### 2.4.4. Thickness Bounds

Considering manufacturing, assembly, and functional requirements, the thickness of each material layer should satisfy

(27) $$h_{i}^{\min}\leq h_{i,j}\leq h_{i}^{\max},\;i=1,\ldots ,L,\;j=1,\ldots ,N.$$

The outer layer should provide sufficient wear resistance, while the middle layer should provide adequate energy absorption. The thickness of the inner layer should also be limited to avoid restricting the joint range of motion.

#### 2.4.5. Total Mass Constraint

The total protective-suit mass should not be too large; otherwise, it increases the robot inertia and affects gait control. Thus,

(28) $$\sum_{j=1}^{N}\sum_{i=1}^{L}\rho_{i}A_{j}h_{i,j}\leq M_{\max},$$

where $M_{\max}$ is the maximum allowable added protective-suit mass.

#### 2.4.6. Total Thickness Constraint

To avoid affecting the range of motion of knees, elbows, hips, shoulders, and other joints, the total thickness of the *j*th region should satisfy

(29) $$\sum_{i=1}^{L}h_{i,j}\leq H_{j}^{\max},\;j=1,\cdots ,N.$$

## 3. Active Protection: VHIP-Based Fall Trajectory Optimization

When a robot predicts an impending fall, it adopts active protection measures to optimize its motion trajectory before the body fully contacts the ground, enabling the robot to enter the contact phase with a lower landing velocity, a lower center of mass height, and a safer posture. When coordinated with passive protection, active protection can significantly reduce a substantial amount of impact energy.

### 3.1. VHIP Model and Fall Detection

**Figure 3:**
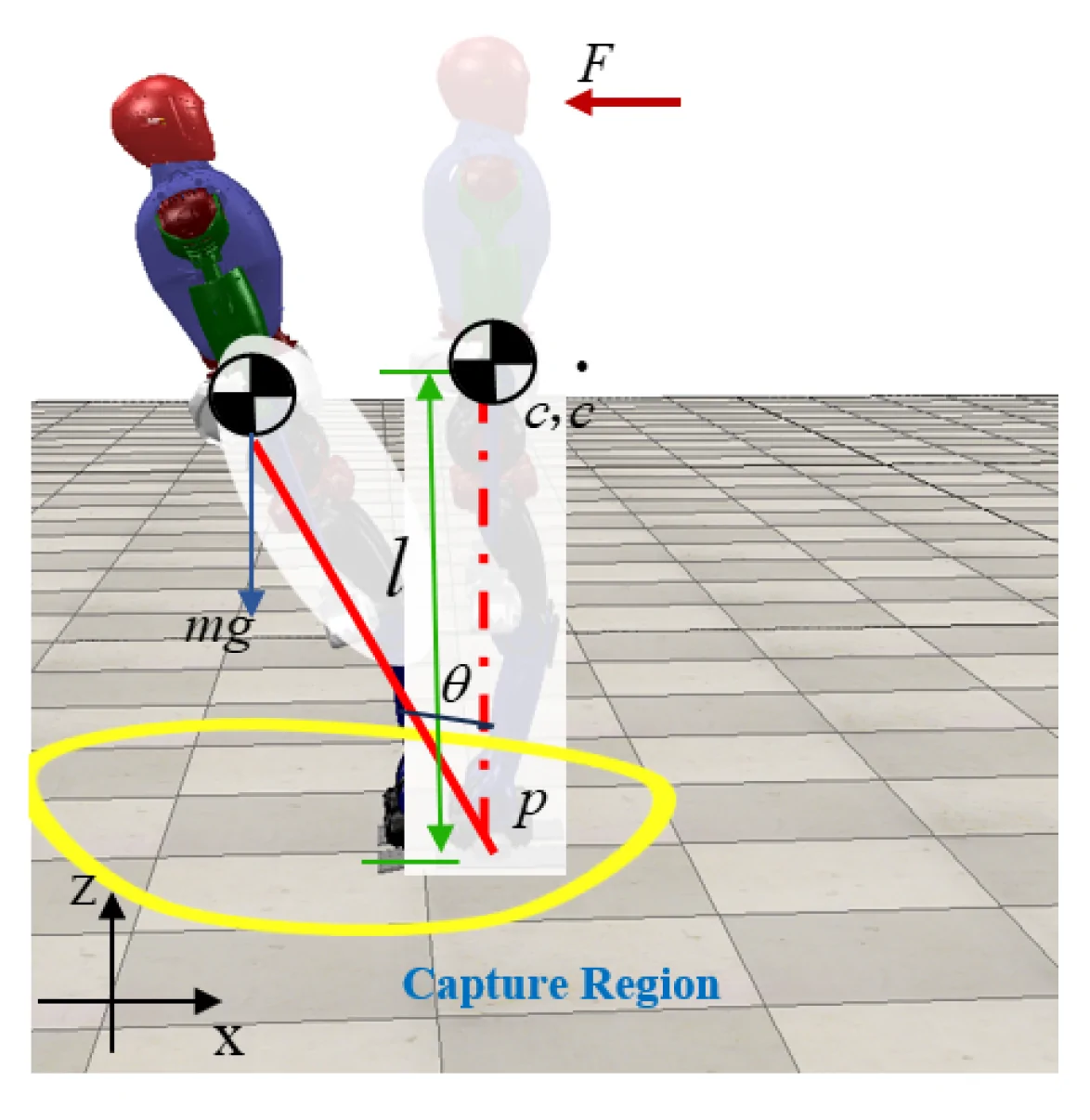
Schematic diagram of the variable-height inverted pendulum model.

A conventional linear inverted pendulum model usually assumes a constant CoM height and can therefore only regulate horizontal CoM motion through the foot CoP. During fall protection, however, the robot often needs to actively lower the CoM height to reduce the landing height and terminal impact energy. Therefore, this paper uses a VHIP model to describe the dominant dynamic characteristics of the robot during a fall. The VHIP model is shown in Figure 3.

Let the CoM position be $\left(c_{x},c_{z}\right)$ and the foot CoP be $p_{x}$. The horizontal dynamics of the VHIP are written as [41]

(30) $${\ddot{c}}_{x}=\omega^{2}\left(c_{x}-p_{x}\right),$$

where

(31) $$\omega^{2}=\frac{g+{\ddot{c}}_{z}}{c_{z}}.$$

Compared with the fixed-height inverted pendulum, ω in the VHIP model is no longer constant; it varies with the CoM height $c_{z}$ and vertical CoM acceleration ${\ddot{c}}_{z}$. Hence, the model can describe both CoP regulation and CoM height regulation, making it more suitable for active fall protection.

To determine whether the robot is difficult to recover, the capture-point concept is introduced following Pratt et al. [3]; its variable-height form is consistent with the VHIP formulation in [41]:

(32) $$\xi_{x}=c_{x}+\frac{{\dot{c}}_{x}}{\omega }.$$

If the capture point has passed the heel boundary with a safety margin,

(33) $$\xi_{x}<x_{\mathrm{heel}}-\Delta_{s},$$

the robot is considered unable to recover standing using conventional balance control alone, and it switches to the fall active protection mode. Here, $x_{\mathrm{heel}}$ is the heel boundary and $\Delta_{s}$ is the safety margin.

### 3.2. Active Protection Optimization Variables

Active protection adopts finite-horizon optimization or model predictive control, with sampling period Δt. The system states are

(34) $$x_{k}=\left[\begin{array}{c} c_{x,k}\\ {\dot{c}}_{x,k}\\ c_{z,k}\\ {\dot{c}}_{z,k} \end{array}\right],$$

and the control variables are

(35) $$u_{k}=\left[\begin{array}{c} p_{x,k}\\ {\ddot{c}}_{z,k} \end{array}\right].$$

Here, $p_{x,k}$ regulates the CoP position, and ${\ddot{c}}_{z,k}$ regulates the CoM height variation. For fall protection, optimizing $p_{x,k}$ helps generate a forward braking acceleration, while optimizing ${\ddot{c}}_{z,k}$ controls squatting or CoM lowering and reduces terminal impact energy.

### 3.3. Discrete Dynamics Model

From the continuous VHIP model,

(36) $${\ddot{c}}_{x,k}=\frac{g+{\ddot{c}}_{z,k}}{c_{z,k}}\left(c_{x,k}-p_{x,k}\right).$$

Using first-order Euler discretization, one obtains

(37) $$c_{x,k+1}=c_{x,k}+\Delta t{\dot{c}}_{x,k},$$

(38) $${\dot{c}}_{x,k+1}={\dot{c}}_{x,k}+\Delta t\frac{g+{\ddot{c}}_{z,k}}{c_{z,k}}\left(c_{x,k}-p_{x,k}\right),$$

(39) $$c_{z,k+1}=c_{z,k}+\Delta t{\dot{c}}_{z,k},$$

(40) $${\dot{c}}_{z,k+1}={\dot{c}}_{z,k}+\Delta t{\ddot{c}}_{z,k}.$$

The above discrete model is used to predict the robot CoM state in the optimization horizon.

### 3.4. Objective Function for Active Protection

Once a fall is judged unrecoverable, the objective of active protection is not to restore the robot to an upright posture, but to guide it toward a controlled landing state that can be safely handled by the passive protective structure. Therefore, the optimization objective is formulated to reduce the horizontal and vertical CoM velocities, lower the CoM height, regulate the control inputs, and minimize the residual energy before contact. Accordingly, the active protection objective function is formulated as:

(41) $$\begin{array}{cc} \min_{{\left\{p_{x,k},\;{\ddot{c}}_{z,k}\right\}}_{k=0}^{N-1}} & J=\sum_{k=0}^{N}\left[w_{1}{\left({\dot{c}}_{x,k}-{\dot{c}}_{x,\mathrm{safe}}\right)}^{2}+w_{2}{\left(c_{z,k}-c_{z,\mathrm{safe}}\right)}^{2}+w_{3}{\dot{c}}_{z,k}^{\;2}\right]\\ \; & \;+\sum_{k=1}^{N-1}w_{4}{\left(p_{x,k}-p_{x,k-1}\right)}^{2}+\sum_{k=0}^{N-1}w_{5}{\ddot{c}}_{z,k}^{\;2}+w_{6}E_{N}^{\mathrm{res}}. \end{array}$$

Here, ${\dot{c}}_{x,\mathrm{safe}}$ is the desired horizontal velocity, and $c_{z,\mathrm{safe}}$ is the desired CoM height before contact. The first three terms regulate the CoM motion throughout the prediction horizon. The fourth term penalizes abrupt variations in the CoP trajectory, while the fifth term limits excessive vertical control effort. The coefficients $w_{1},\ldots ,w_{6}>0$ are weighting factors.

The residual energy at the end of the prediction horizon is approximated as

(42) $$E_{N}^{\mathrm{res}}=\frac{1}{2}m_{\mathrm{eff}}\left({\dot{c}}_{x,N}^{\;2}+{\dot{c}}_{z,N}^{\;2}\right)+m_{\mathrm{eff}}g\left[c_{z,N}-c_{z,\mathrm{contact}}\right],$$

where $m_{\mathrm{eff}}$ is the equivalent impact mass, and $c_{z,\mathrm{contact}}$ is the reference CoM height at the beginning of the main contact phase. The terminal energy term encourages the optimizer to generate a low velocity and low height state that can be safely absorbed by the passive protective layers.

### 3.5. Active Protection Constraints

#### 3.5.1. Contact Feasibility Constraints

To guarantee contact stability and physical feasibility during the fall protection process, the CoP trajectory and ground interaction forces are constrained within their allowable regions [45]. During the fall protection process, the CoP must remain inside the available foot-support region. Therefore, the sagittal CoP position is constrained as

(43) $$p_{x}^{\min}\leq p_{x,k}\leq p_{x}^{\max},\;k=0,\ldots ,N-1,$$

where $p_{x}^{\min}$ and $p_{x}^{\max}$ denote the rear and front boundaries of the support region, respectively. For fall protection, the optimized CoP is expected to move toward the heel side so as to generate a braking effect against the rotation.

To avoid foot slipping, the ground reaction force must satisfy the friction-cone constraint

(44) $$|F_{x,k}|\leq \mu F_{z,k},$$

where μ is the ground friction coefficient. Using the VHIP dynamics, this constraint can be written as

(45) $$|{\ddot{c}}_{x,k}|\leq \mu \left(g+{\ddot{c}}_{z,k}\right).$$

Since

(46) $${\ddot{c}}_{x,k}=\frac{g+{\ddot{c}}_{z,k}}{c_{z,k}}\left(c_{x,k}-p_{x,k}\right),$$

the friction constraint can be equivalently expressed as

(47) $$|c_{x,k}-p_{x,k}|\leq \mu c_{z,k}.$$

#### 3.5.2. CoM Motion Feasibility Constraints

The CoM height cannot be reduced arbitrarily because of joint range, self collision, and actuation limitations. Thus, the CoM height is bounded by

(48) $$c_{z}^{\min}\leq c_{z,k}\leq c_{z}^{\max},\;k=0,\ldots ,N.$$

In addition, the vertical acceleration of the CoM is limited by the actuation capability and the feasible squatting motion of the humanoid robot:

(49) $${\ddot{c}}_{z}^{\min}\leq {\ddot{c}}_{z,k}\leq {\ddot{c}}_{z}^{\max},\;k=0,\ldots ,N-1.$$

These two constraints prevent the optimizer from generating unrealistically aggressive height variations during the short falling process.

#### 3.5.3. Terminal Safety and Energy Matching Constraints

At the end of the active protection phase, the robot should enter a low CoM and low impact state before the main body ground contact occurs. Therefore, the terminal CoM state is constrained as

(50) $$c_{z,N}\leq c_{z,\mathrm{safe}},\;\left|{\dot{c}}_{z,N}\right|\leq {\dot{c}}_{z,\mathrm{safe}}.$$

To couple active protection with passive structural absorption, the residual impact energy after active protection must not exceed the safe absorption capacity of the passive protective layers:

(51) $$E_{N}^{\mathrm{res}}\leq E_{\mathrm{pass}}^{\mathrm{safe}}.$$

The safe passive absorption capacity is determined by the selected high-risk contact regions involved in falling:

(52) $$E_{\mathrm{pass}}^{\mathrm{safe}}=\frac{1}{k_{E}}\sum_{j\in P_{\mathrm{back}}}E_{j}^{\mathrm{abs}},$$

where $P_{\mathrm{back}}$ denotes the set of primary contact regions during a fall, $E_{j}^{\mathrm{abs}}$ is the energy-absorption capacity of the passive layer in the *j*th region, and $k_{E}$ is the safety factor.

These constraints ensure that the active protection module does not merely generate a feasible falling motion, but also produces a terminal state that can be safely handled by the passive protective structure.

## 4. Simulation

The simulation procedure implements the methods developed in Section 2 and Section 3 in a sequential manner. First, the multi-directional falling responses are used in the risk-evaluation model of Section 2 to identify the key body regions requiring protection. The multilayer thicknesses of these regions are subsequently optimized using Equation (16) and the associated constraints. Second, when the fall-prediction criterion in Section 3 indicates that balance recovery is no longer feasible, the VHIP-based active-protection optimization is activated. The CoM motion is predicted using Equations (36)–(40), and the CoP position and vertical CoM acceleration are optimized according to Equation (41). Finally, four cases—no protection, passive protection only, active protection only, and passive–active cooperative protection—are simulated under the same disturbance condition to evaluate the individual and combined effects of the two protection mechanisms.

**Figure 4:**
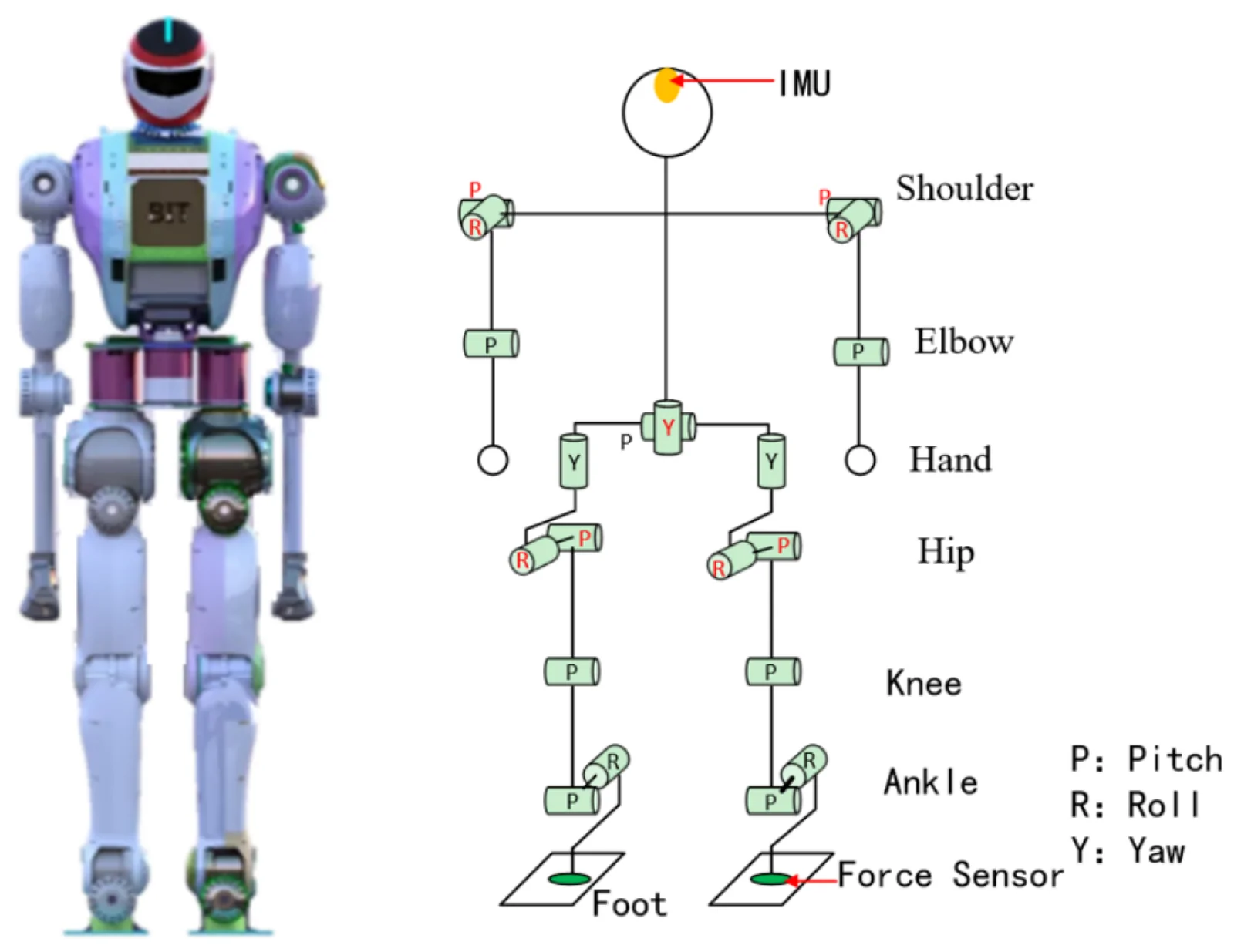
Snapshots of the humanoid robot. The left side shows the three-dimensional robot model, and the right side shows the schematic diagram of the robot joints.

The validation is conducted on the FCR humanoid robot, as shown in Figure 4; the FCR robot is a full-body humanoid platform consisting of a torso, waist, two arms, and two legs, with its main dimensional and physical parameters summarized in Table 1. The robot has a mass of approximately 50 kg, a standing height of approximately 165 cm, and 22 degrees of freedom, including six degrees of freedom in each leg, two in the waist, and four in each arm. In the simulation, the robot is controlled in position-control mode. In this paper, a co-simulation platform is constructed based on MATLAB R2023a and CoppeliaSim version 4.1, where the robot adopts the same position control mode as the actual FCR robot.

### 4.1. Passive Protection Contact Region Identification

**Figure 5:**
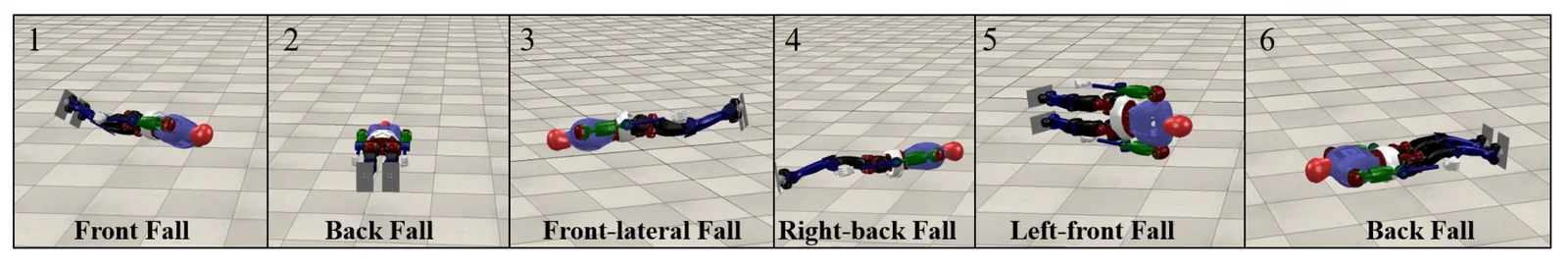
Multi-directional fall simulations used for contact-region risk evaluation.

Passive protection is not implemented by uniformly adding protective layers to the entire robot body. Instead, the body regions that are most likely to contact the ground and suffer high impact loads during falling are first identified. In the simulation, the robot is initially placed in a natural standing posture, and external disturbance forces are applied near the trunk, shoulder, or hip to induce different falling scenarios. The disturbance magnitude is set to 500 N, while the disturbance direction is varied according to the prescribed fall direction.The representative falling behaviors of the robot under different disturbance directions are shown in Figure 5, and the corresponding risk-evaluation parameters are summarized in Table 2. Robot-related quantities are obtained from the FCR humanoid robot model and multi-directional fall simulations, whereas material-related parameters are determined from the physical properties of the protective materials. The parameters listed in Table 2 are design parameters selected by the authors according to the requirements of impact-risk evaluation and protection optimization.

To calculate the force-related risk term in the regional risk index, the allowable peak impact-force thresholds for the candidate body regions were determined specifically for the FCR humanoid robot based on engineering experience and manufacturer-provided information regarding its structural characteristics, joint properties, contact areas, and internal component distribution. Lower allowable thresholds were assigned to relatively vulnerable regions, whereas higher thresholds were assigned to structurally stronger regions, as summarized in Table 3.

According to the above risk-evaluation method, the chest, back, hip, waist, shoulder, elbow, knee, upper arm, forearm, and sole are screened as key protective regions in the simulation. These regions have high contact probability or high impact risk in multi-directional fall simulations. Therefore, the subsequent thickness optimization mainly focuses on these regions.

### 4.2. Passive Protective Layer Modeling and Thickness Optimization

In the passive protection design, a composite protective suit with multiple protected regions and three material layers (L=3) is developed for the humanoid robot. Unlike a uniform thick shell, the protective layers are applied only to the key body regions identified through the preceding risk assessment. In each protected region, the outer silicone layer provides wear and scratch resistance and distributes the contact load through compliant deformation. The middle sponge layer provides a large compression stroke, thereby extending the impact duration and reducing the peak impact force. The inner foam layer conforms to the robot shell and absorbs the remaining energy, reducing the impact transmitted to the internal structure.

**Figure 6:**
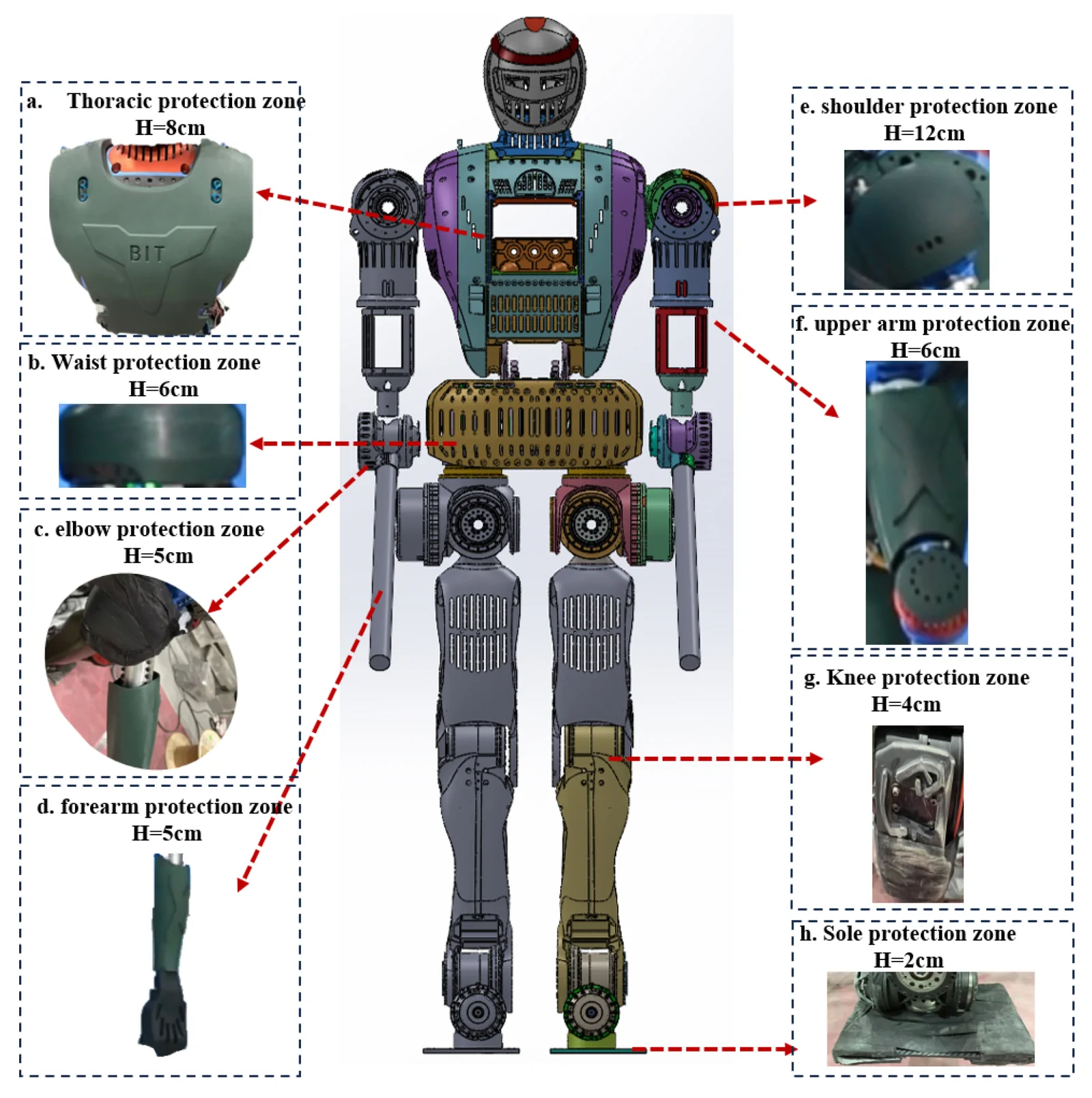
Protective regions and optimized thickness distribution for the humanoid robot.

After the key protective regions are identified using $E_{0,j}^{\max}$, $F_{j}^{\max}$, $v_{j}^{\max}$, and $P_{j}$, the design impact energy of each region is calculated as $E_{0,j}=\kappa_{E}E_{0,j}^{\max}$. The material properties, protected area, allowable impact force, and prescribed safety factors are then used to formulate the constraints on energy absorption, peak impact force, material densification, total mass, and total thickness. The resulting quadratic programming problem is solved using the MATLAB quadprog solver to obtain the optimal thickness $h_{i,j}^{*}$ of each material layer in every protected region. The optimized values are further adjusted according to manufacturing tolerances and available material specifications, and the final thickness distribution is shown in Figure 6.

### 4.3. Performance Comparison of Protection Schemes

**Figure 7:**
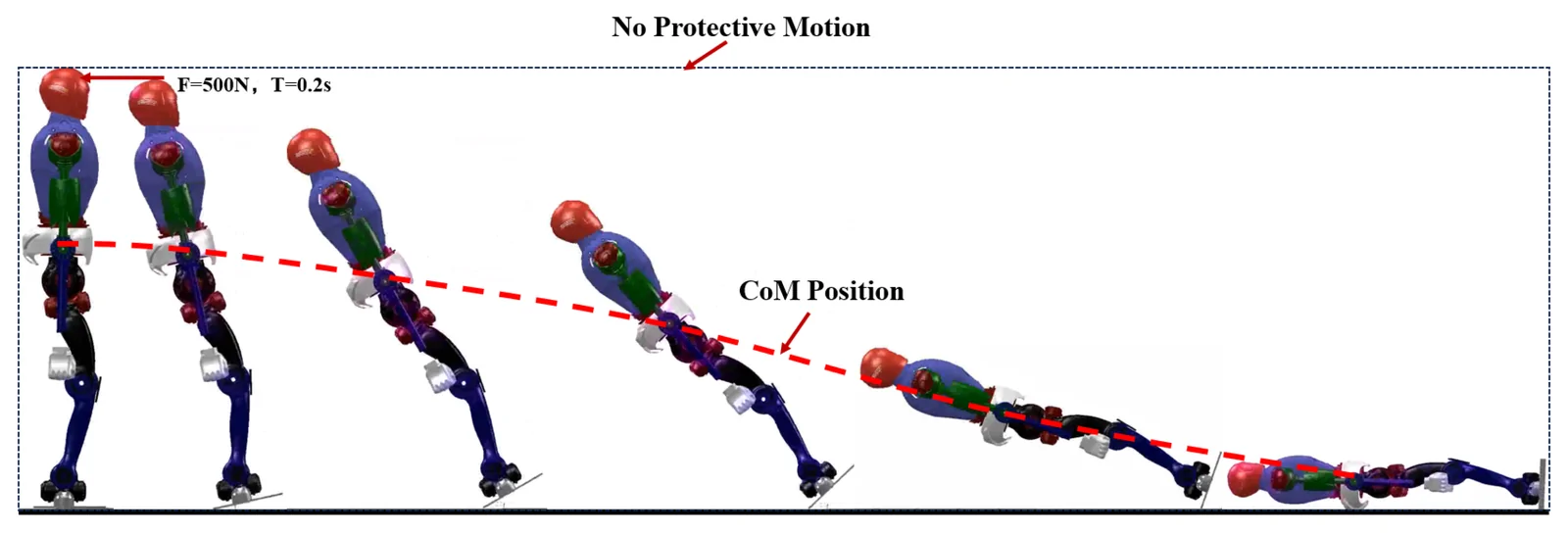
No protection falling strategy.

**Figure 8:**
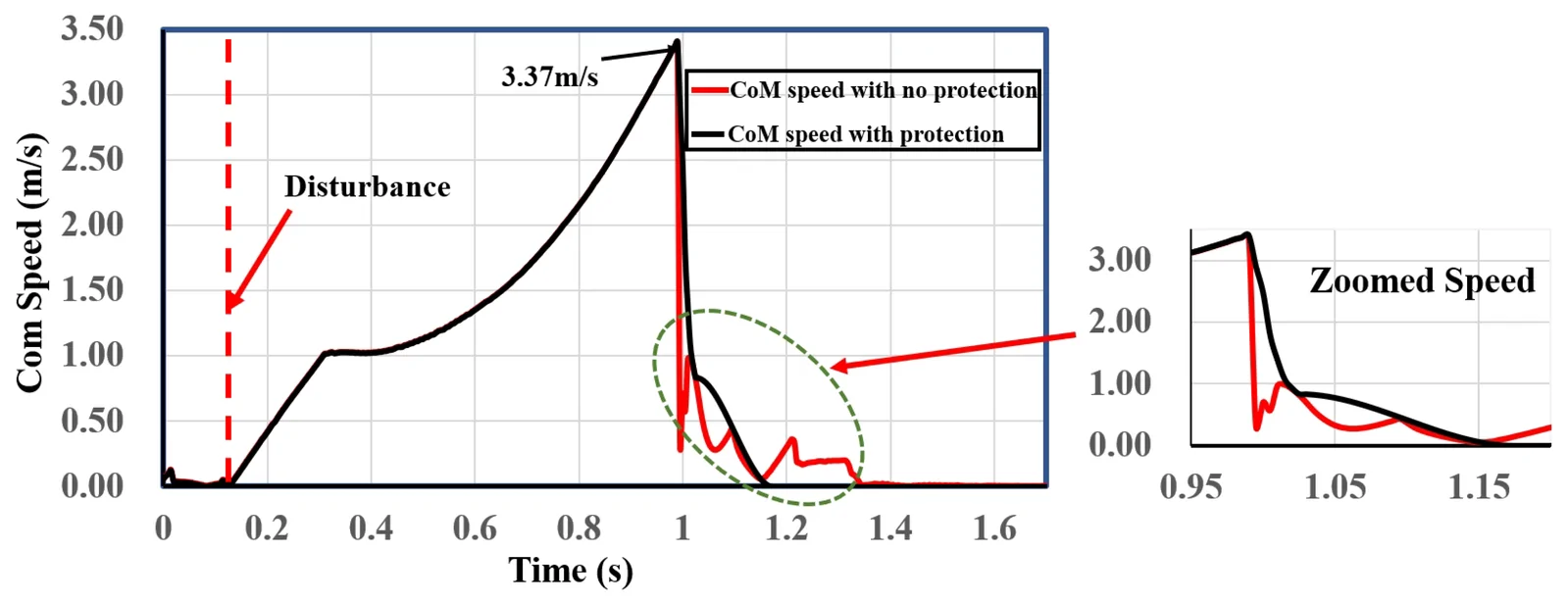
CoM speed response under the same 500 N, 0.2 s backward disturbance: no protection versus passive protection.

**Figure 9:**
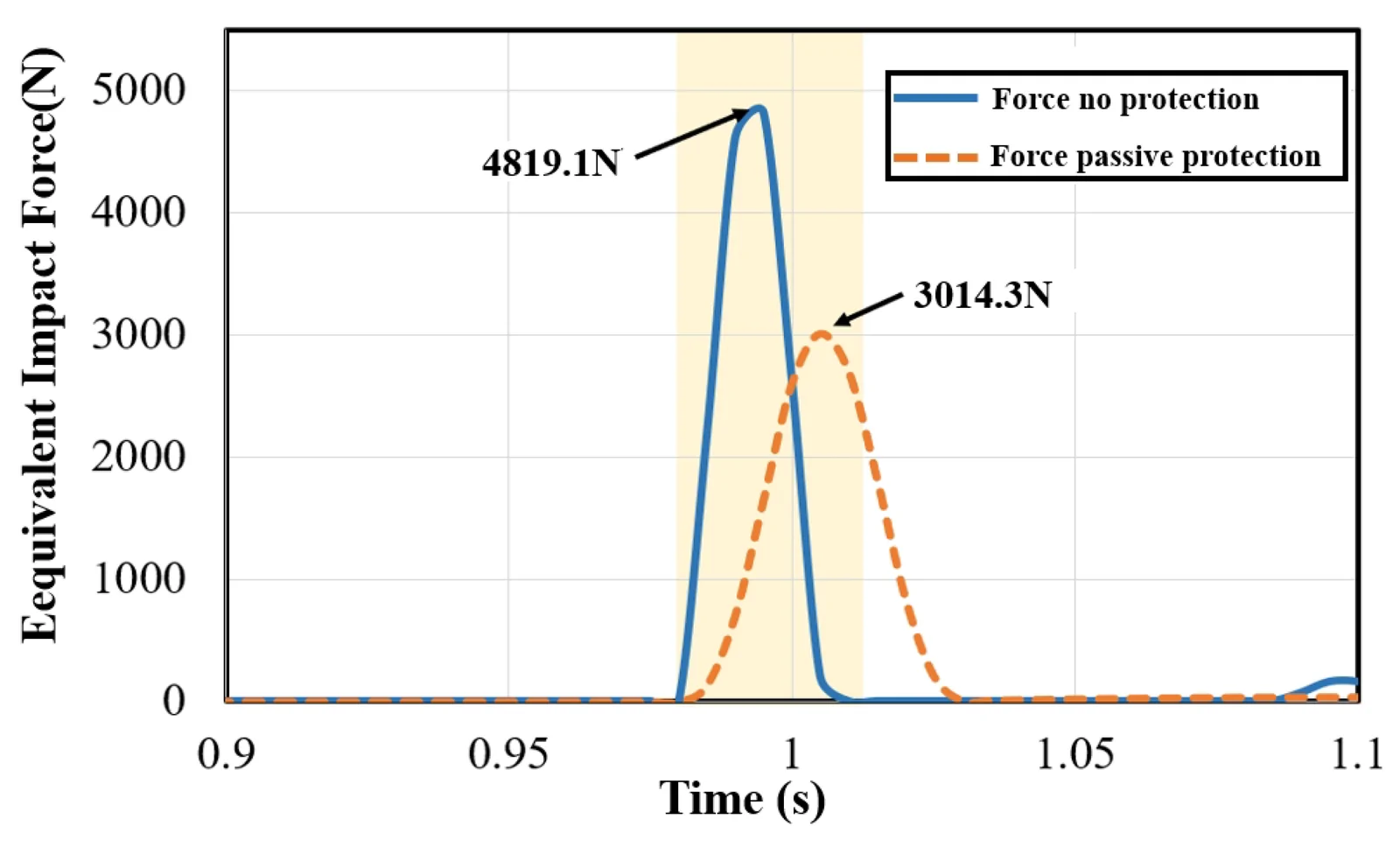
Equivalent impact-force response under the same disturbance: no protection versus passive protection.

To verify the effectiveness of the proposed method, backward falls are adopted for validation in this paper. Compared with other fall patterns, backward falls produce the largest impact force, which justifies their use as the validation scenario. Four working conditions are configured in the simulation: no protection, passive protection only, active protection only, and cooperative passive and active protection. In the no protection case, the robot starts from a natural standing posture and is subjected to a disturbance force of 500 N for 0.2 s, as illustrated in Figure 7. Since no protective strategy is activated, the robot loses balance under the disturbance, develops an irrecoverable falling motion, and finally impacts the ground directly. For a fair comparison among the four cases, the same initial standing posture, the same 500 N disturbance applied for 0.2 s, and the same ground-contact conditions are used. Therefore, the differences observed in Figure 8, Figure 9 and Figure 11, Figure 12 and Figure 13 are attributed to the activated protection strategy rather than to a change in the external disturbance.

Because most robot simulation platforms mainly target multi-rigid-body motion and rigid contact simulation, it is difficult to directly and accurately describe the nonlinear compression, damping dissipation, and interlayer coupling of soft materials such as silicone, sponge, and foam during impact. Therefore, in this simulation, the multi-layer protective structure is equivalently modeled as a Kelvin–Voigt spring–damper element to validate the effectiveness of the method. When the protective layer is compressed, the normal contact force is expressed as [46]

(53) $$F_{p}=k_{\mathrm{eq}}\delta +c_{\mathrm{eq}}\dot{\delta },\;\delta >0,$$

where δ and $\dot{\delta }$ are the compression displacement and compression velocity of the protective layer, respectively, and $k_{\mathrm{eq}}$ and $c_{\mathrm{eq}}$ are the equivalent stiffness and equivalent damping coefficients, respectively. Under the same initial posture, external disturbance, and ground-contact conditions, no-protection and passive protection cases are compared.

During the co-simulation, the robot CoM motion is continuously recorded in CoppeliaSim, and the corresponding time-domain CoM speed responses are extracted for comparison, as shown in Figure 8. Under the same 500 N disturbance, the no-protection and passive-protection curves almost overlap throughout the pre-contact falling phase and reach the same maximum CoM speed of approximately 3.37 m/s. This agreement confirms that the passive layer does not modify the disturbance-induced falling motion before contact; instead, it becomes effective only when the protected body region touches the ground. After contact, the no-protection case shows an abrupt velocity drop followed by pronounced rebound and oscillation, whereas the passive-protection case exhibits a smoother deceleration over a longer duration and a smaller rebound. Thus, Figure 8 shows that passive protection mainly acts in the post-contact stage by increasing the effective stopping distance and dissipating residual kinetic energy rather than by changing the pre-impact trajectory.

The robot’s ground impact-force response is recorded during the same backward-fall disturbance case. In the no-protection case, the contact force is obtained directly from the rigid robot–ground contact simulation. In the passive-protection case, the multilayer protective structure is represented by the equivalent Kelvin–Voigt model, and the compliant contact force is calculated using Equation (53). The resulting time-domain responses are compared in Figure 9. Because the disturbance input and pre-contact CoM speed are the same in the two cases, the difference in the force response directly reflects the effect of the passive protective layer. The peak equivalent impact force decreases from 4819.1 N without protection to 3014.3 N with passive protection, corresponding to a reduction of approximately 37.5%. Meanwhile, the protected case exhibits a wider force pulse and a slightly delayed peak. In conjunction with Figure 8, this result indicates that the passive layer does not reduce the approach speed, but converts the short-duration rigid impact into a longer-duration compliant contact process, thereby lowering the instantaneous peak load through elastic deformation and damping dissipation.

Based on the no-protection and passive-protection cases, active protection and passive–active cooperative protection are further tested. For the active-protection optimization, the sagittal CoP position, CoM height, and vertical CoM acceleration are constrained as $p_{x,k}\in \left[-0.08,\;0.10\right]\;m$, $c_{z,k}\in \left[0.18,\;0.95\right]\;m$, and ${\ddot{c}}_{z,k}\in \left[-10.0,\;10.0\right]\;m/s^{2}$, respectively. These bounds are selected according to the available foot-support region, feasible CoM motion range, and actuation capability of the humanoid robot. The active-protection trajectory optimization parameters are set as $c_{z,\mathrm{safe}}=0.18\;m$, ${\dot{c}}_{z,\mathrm{safe}}=0\;m/s$, $E_{\mathrm{safe}}=50\;J$, and $p_{x,\mathrm{ref}}=-0.09\;m$. The target CoM height in the contact phase is set as $c_{z,\mathrm{contact}}=0.09\;m$.

**Figure 10:**
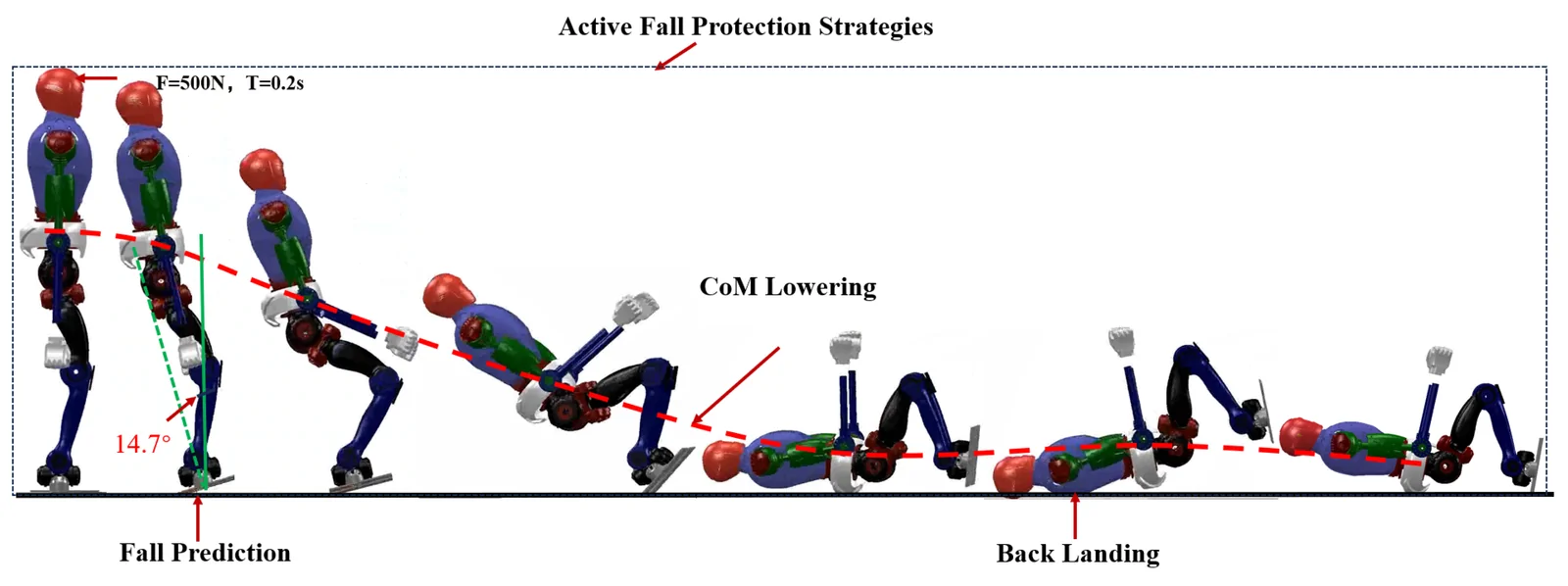
Active protection strategy for falling.

The backward-fall process of the robot under coordinated active and passive protection is shown in Figure 10. The robot initially keeps a standing posture, and the fall-prediction module continuously monitors its motion state. When the trunk inclination exceeds 14.70°, and according to the capture point theory introduced above, the system judges that the robot has entered an unrecoverable fall and triggers active protection trajectory optimization based on the VHIP model. The robot actively lowers the CoM by flexing the hip and knee joints, thereby reducing the impact energy before landing. Considering that the robot hands contain precision finger mechanisms, the controller drives both arms forward to avoid direct hand-ground collision. The target forearm joint angle is set to 80°, and a smooth joint trajectory is generated using spline interpolation. Finally, the robot contacts the ground mainly through the back region.

**Figure 11:**
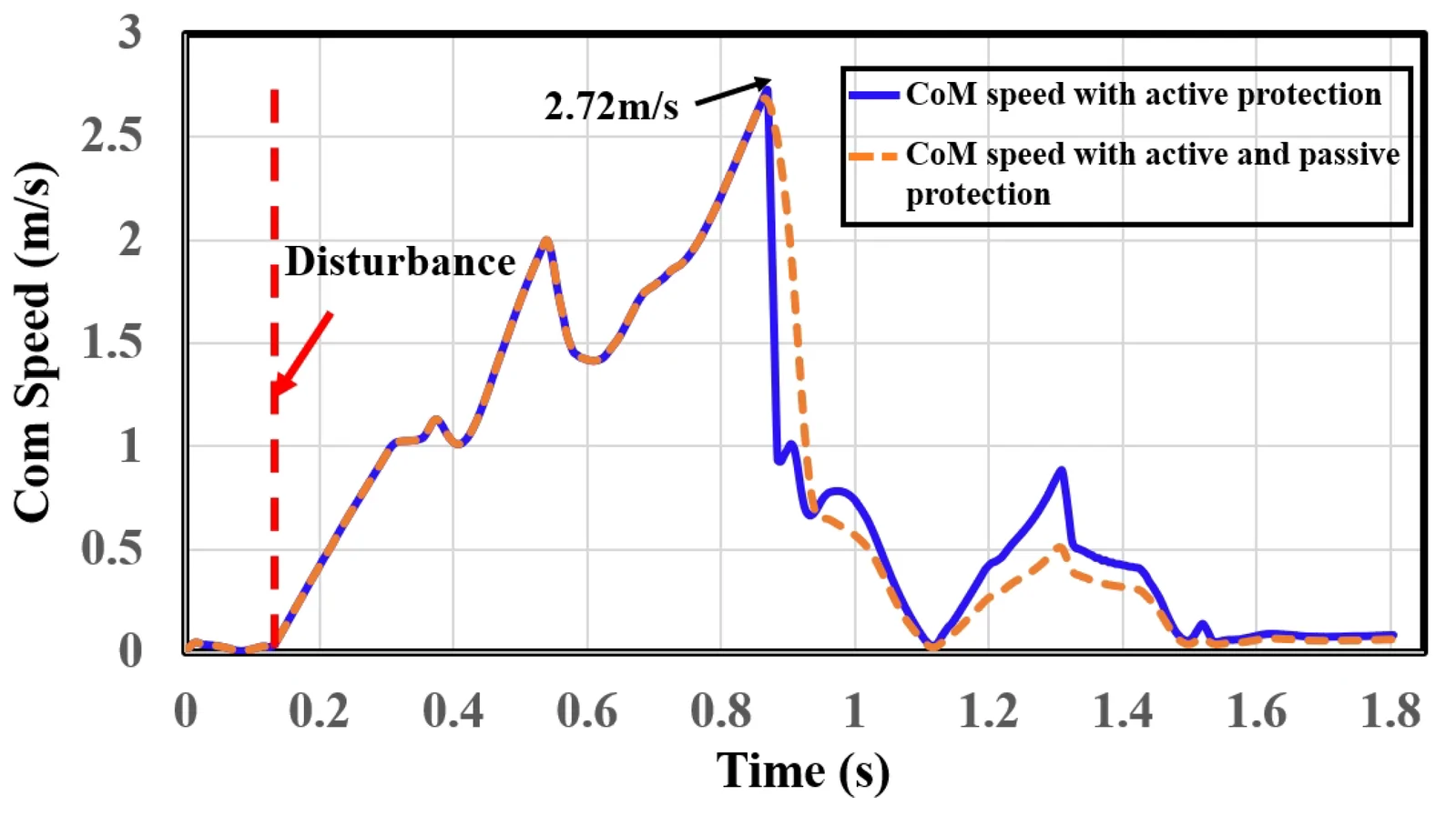
CoM speed response under the same disturbance: active protection versus passive–active cooperative protection.

Figure 11 compares the CoM speed for the active-protection and passive–active cooperative-protection cases under the same 500 N, 0.2 s disturbance. Before ground contact, the two curves almost completely overlap because both cases execute the same active fall-protection trajectory, and the maximum CoM speed is approximately 2.72 m/s. Compared with the 3.37 m/s maximum speed in the no-protection/passive-only cases in Figure 8, active trajectory regulation reduces the maximum pre-contact CoM speed by approximately 19.3%. This confirms that active protection primarily modifies the disturbance-induced falling state before impact by lowering the CoM and regulating the landing posture. After contact, the cooperative-protection curve exhibits smaller rebound and lower post-impact oscillation than the active-only curve. Therefore, the two protection mechanisms have complementary roles: active control reduces the pre-impact kinetic state, whereas the passive layer dissipates the remaining impact energy after contact.

**Figure 12:**
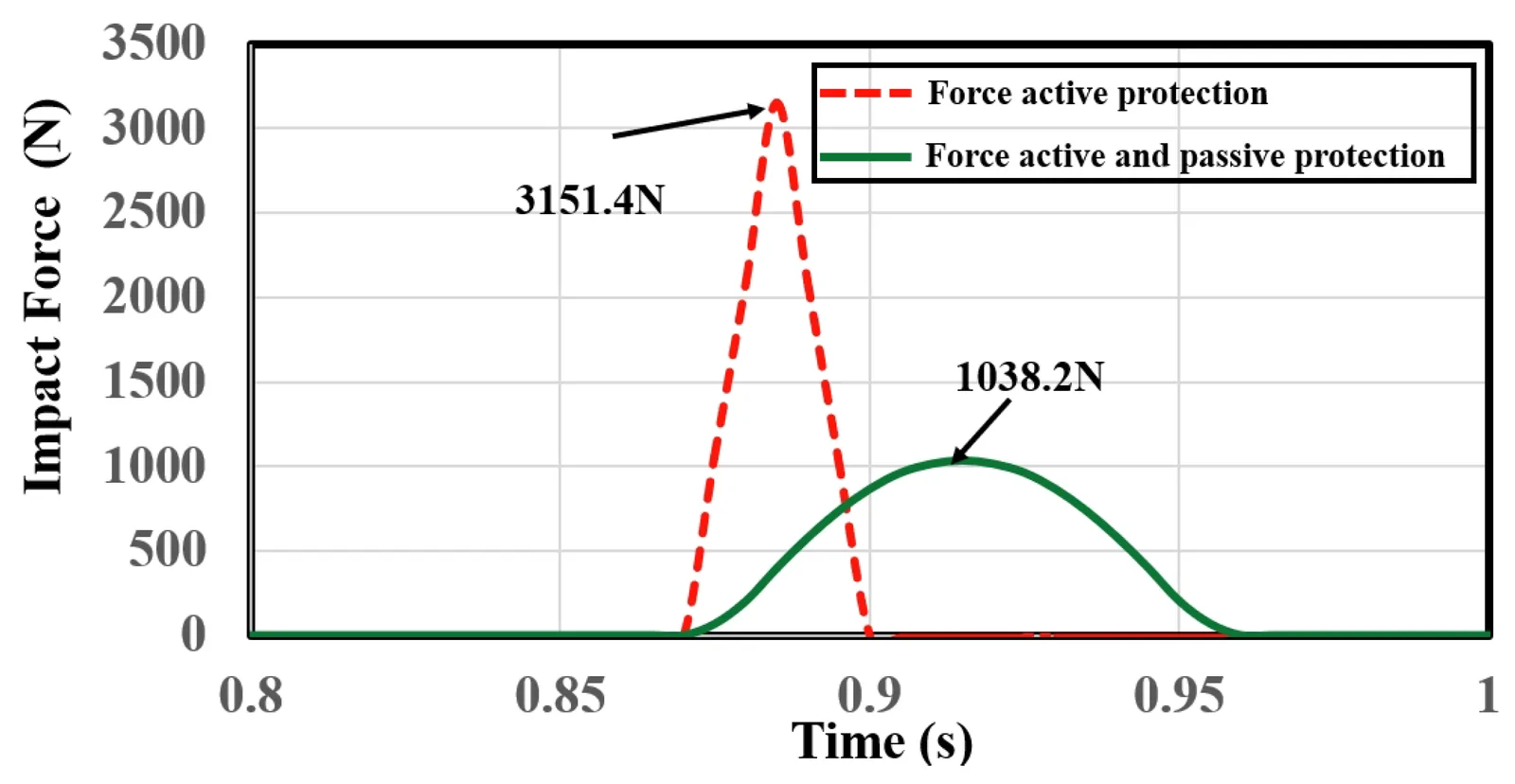
Equivalent impact-force response under the same disturbance: active protection versus passive–active cooperative protection.

Figure 12 further compares the impact-force responses of the active-only and cooperative-protection cases under the same disturbance. With active protection alone, the peak equivalent impact force is 3151.4 N, which is approximately 34.6% lower than the 4819.1 N peak of the no-protection case in Figure 9; this reduction is mainly attributed to the lower pre-contact CoM speed produced by the active trajectory. When passive protection is combined with active control, the peak force further decreases to 1038.2 N, corresponding to a 67.1% reduction relative to active protection alone and an overall reduction of approximately 78.5% relative to the no-protection case. The cooperative curve also exhibits a broader and smoother force pulse. These results demonstrate that active control and passive cushioning act sequentially on different phases of the same disturbance-induced fall and together provide substantially greater impact attenuation than either mechanism acting alone.

**Figure 13:**
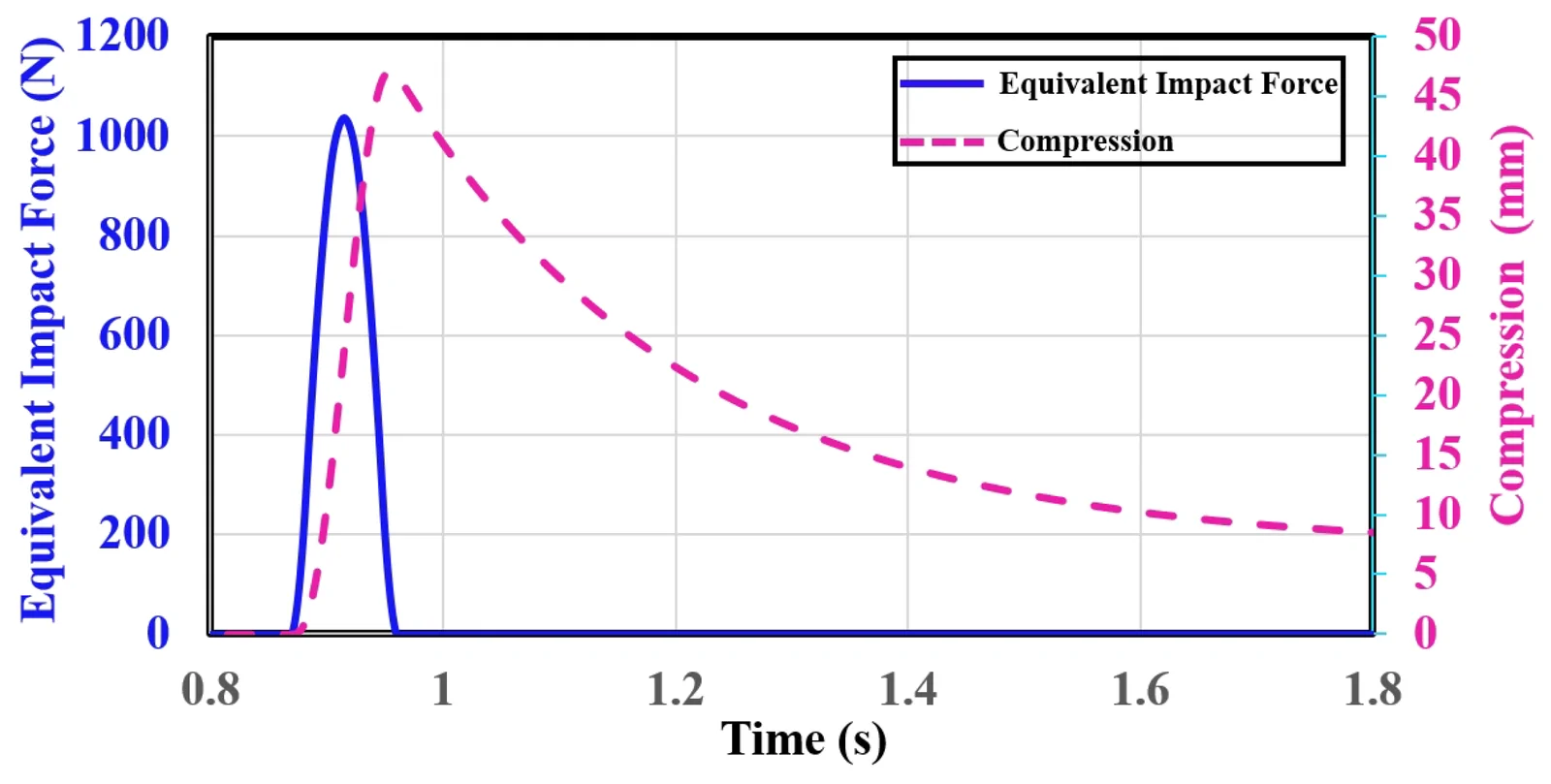
Equivalent impact-force and protective-layer compression responses in the passive–active cooperative protection case.

Figure 13 explains the contact-stage mechanism responsible for the cooperative-protection result in Figure 12. After the disturbance has triggered the fall and the active trajectory has reduced the pre-impact state, the back protective layer is compressed when ground contact occurs. The equivalent impact force rises rapidly and reaches a peak consistent with the cooperative-protection force in Figure 12; the compression reaches its maximum shortly afterward and then recovers gradually. This temporal offset between force and compression is consistent with the spring–damper behavior of the equivalent protective layer: part of the residual impact energy is temporarily stored through elastic deformation, while another part is dissipated through damping. The gradual recovery of the compression also suppresses an abrupt secondary rebound. Hence, Figure 13 provides a mechanical explanation for the lower and smoother impact-force response observed in the cooperative case.

Overall, Figure 8, Figure 9, Figure 10, Figure 11, Figure 12 and Figure 13 reveal the complete disturbance–protection sequence. The external disturbance first drives the robot into an unrecoverable backward fall. Active protection acts before contact and reduces the maximum CoM speed from approximately 3.37 m/s to 2.72 m/s, while passive protection acts mainly after contact and reduces the peak load by extending the collision duration. When the two mechanisms are combined, the peak impact force decreases from 4819.1 N in the no-protection case to 1038.2 N, i.e., by approximately 78.5%, while post-impact rebound is also reduced. These comparisons clarify the respective and complementary contributions of disturbance response, active motion regulation, and passive energy absorption in the proposed cooperative protection strategy.

## 5. Experiment

**Figure 14:**
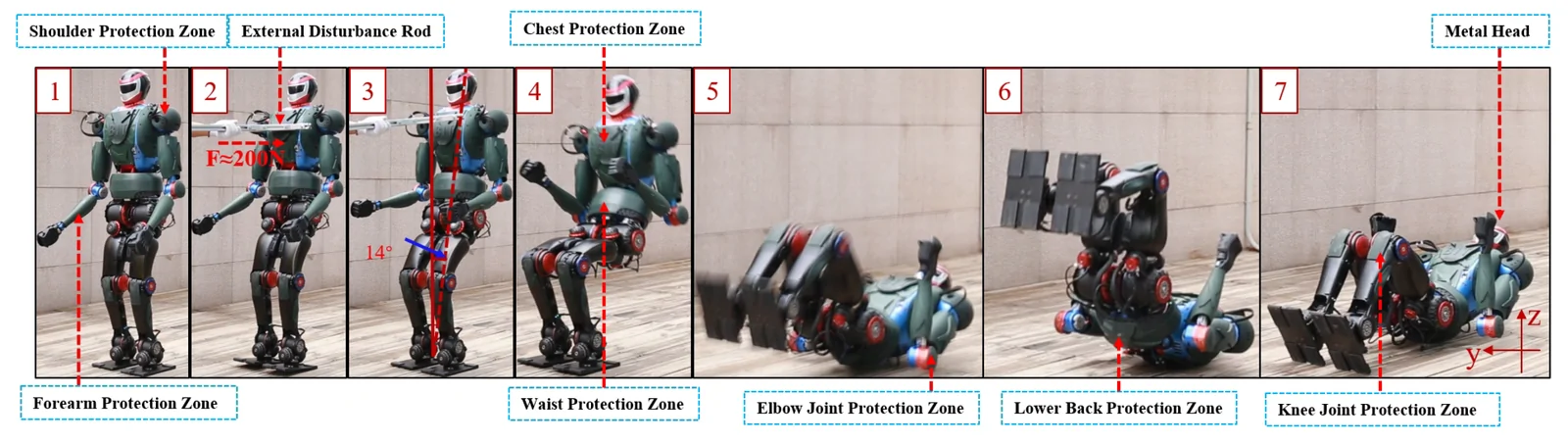
Physical experiment of passive active cooperative fall protection.

To verify the feasibility of the proposed passive active cooperative fall protection method on a real robot, physical experiments are conducted on the FCR robot. As shown in Figure 14, cushioning protection devices are installed in key regions including the shoulders, forearms, chest, waist, back, elbows, and knees. The robot initially maintains a standing posture. A horizontal push of approximately 200 N is then applied to the upper trunk to make the robot lose balance. During the experiment, the fall-prediction and protection-control program runs continuously, and the robot falling posture and CoM position are recorded.

The experimental sequence shows that the robot initially keeps a natural standing posture. After the external push, the trunk gradually tilts. When the inclination reaches approximately 14.0°, the robot’s capture point exceeds the predefined stability margin, and the fall-protection strategy is triggered. The robot then flexes the hip and knee joints to actively lower the CoM while driving both arms forward to avoid direct impact on the precision hand structures. Subsequently, the robot mainly contacts the ground through the rear waist, back, and elbows in sequence. The head approaches the ground last, and the robot finally forms a stable supine posture while the knees remain flexed. No obvious lateral rolling occurs during this process, indicating that the designed active protection motion can be stably executed on the real robot and that the passive protective devices cover the main contact regions.

**Figure 15:**
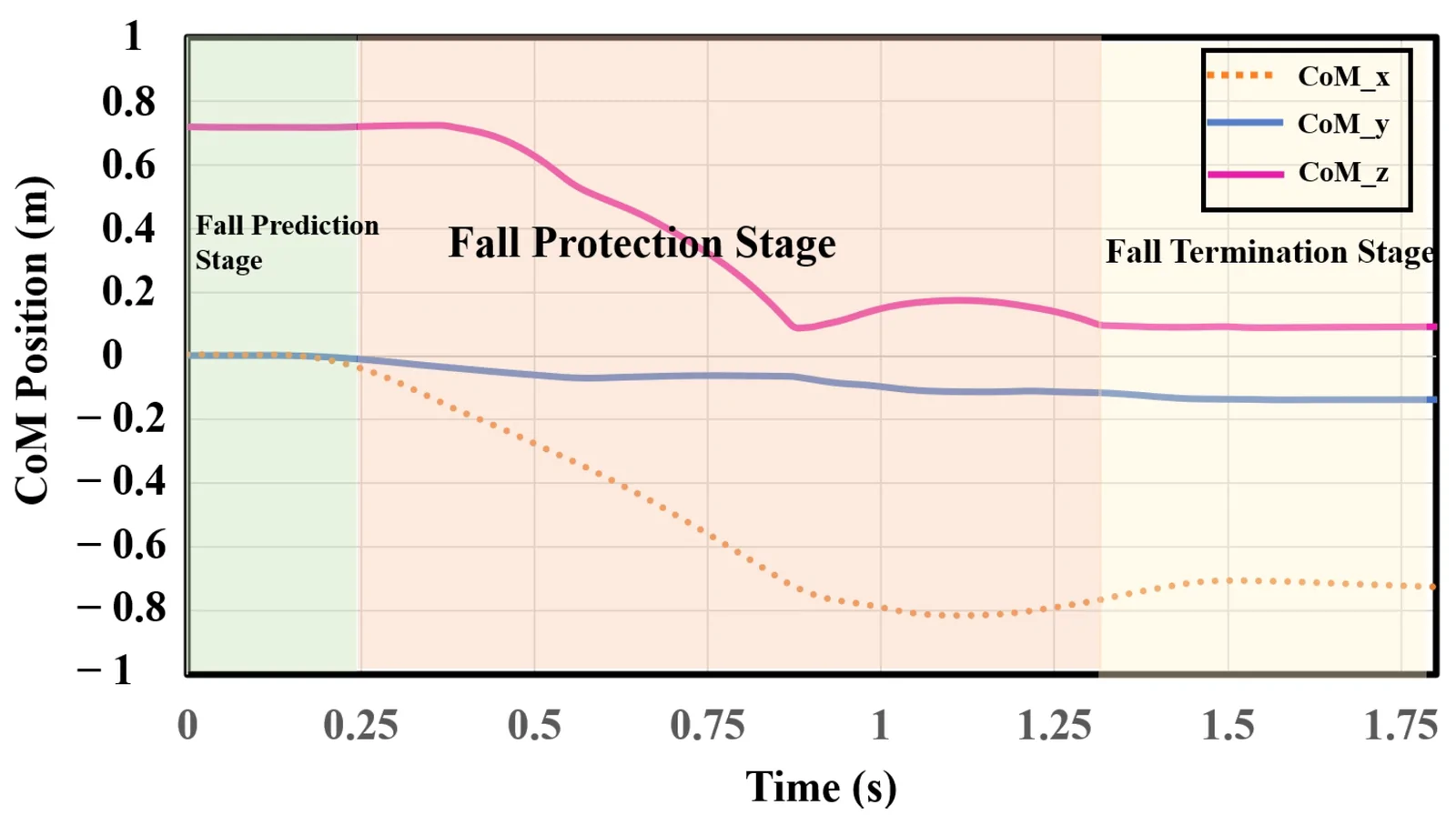
CoM position variation of the robot in the physical experiment.

Figure 15 shows the variations of the robot CoM position in the *x*, *y*, and *z* directions during the physical experiment. According to the motion state, the process can be divided into three stages: fall prediction, fall protection, and fall termination. In the fall-prediction stage, the CoM gradually deviates from the standing equilibrium region. In the fall-protection stage, active joint motion lowers the vertical CoM height and guides the body posture toward a safer contact configuration. In the fall-termination stage, the passive protective layers dissipate the residual impact energy, and the CoM variation gradually becomes stable. These results show that the proposed cooperative strategy can reduce the violent impact at landing and improve the safety of physical humanoid robot falls.

**Figure 16:**
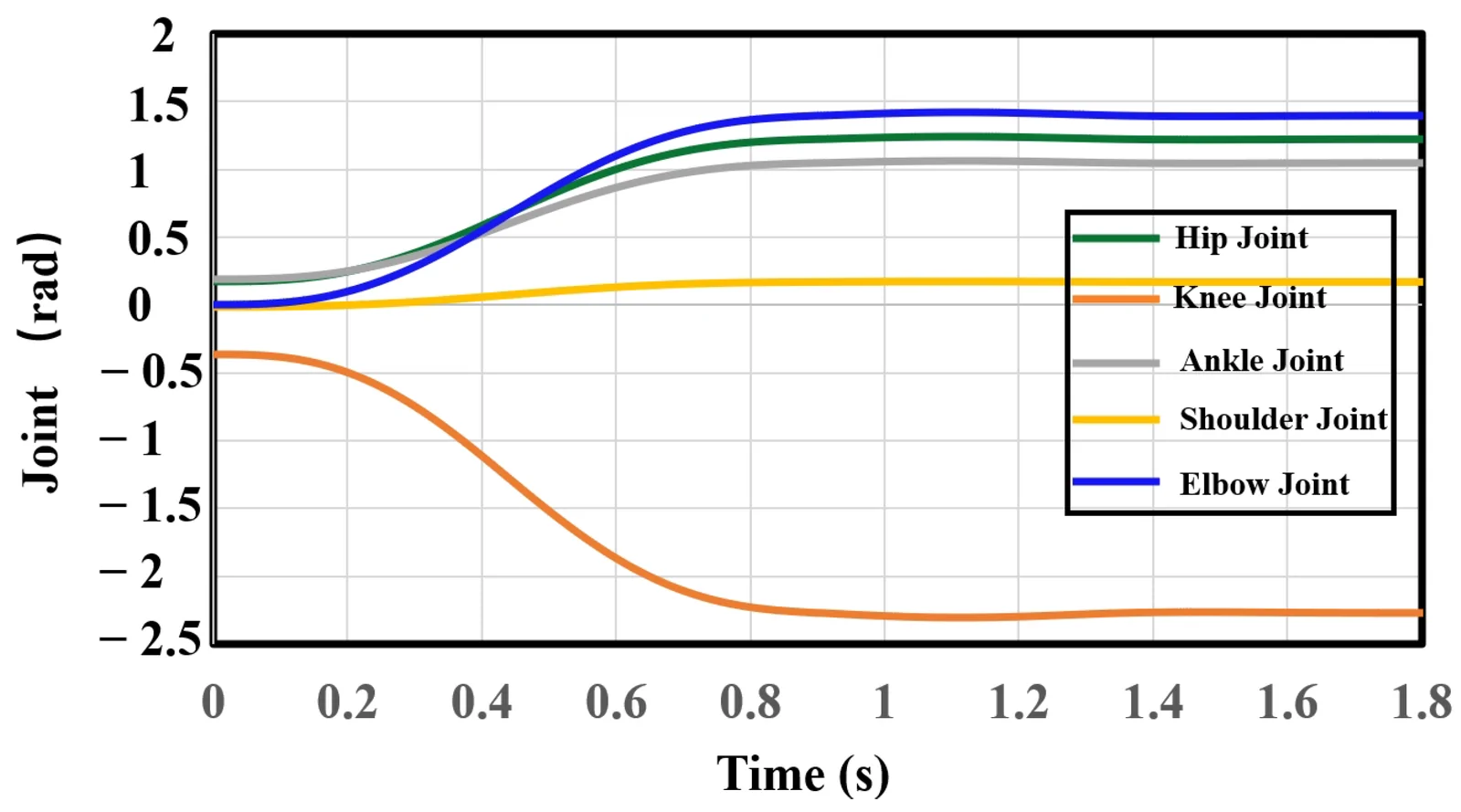
Robot joint positions.

Meanwhile, the robot joint positions were recorded, as shown in Figure 16. The joint trajectories are generally smooth and continuous, without obvious abrupt changes or discontinuities. It should be noted that the negative knee-joint angle results from the joint-coordinate convention of the FCR robot, in which the knee-flexion direction is defined as negative. Therefore, the negative value represents normal knee flexion rather than reverse bending. During the fall-protection motion, the knee flexes to lower the CoM, and its trajectory remains within the allowable mechanical joint range.

## 6. Conclusions

This paper proposed a cooperative passive and active method for humanoid robot fall protection. Key protective regions were identified through simulations of falls in multiple directions, and the multilayer thicknesses were optimized under protection and lightweight constraints. A VHIP model was used to regulate the CoP and CoM trajectories, while the residual energy after active protection was matched with the absorption capacity of the passive layers. In the backward fall simulation, passive protection reduced the peak equivalent impact force by 37.5%, and the cooperative method further reduced it to 1038.2 N, representing a 78.5% reduction compared with no protection. Physical experiments on the FCR robot confirmed that the protective motion could be executed smoothly and that the robot reached a stable supine posture. Future work will further conduct systematic experimental validation of the proposed protection framework under complex falling postures and different ground conditions.

## Figures and Tables

**Table 1:** Parameters of the FCR robot.

Parameter	Size	Mass
Thigh	361 mm	7.36 kg
Shank	330 mm	5.12 kg
Boom	350 mm	4.15 kg
Jib	360 mm	2.30 kg
Others	—	31.07 kg
Total mass	—	50.00 kg

**Table 2:** Risk-evaluation parameter settings.

Parameter	Value
κ	1.5
$\Delta t_{j,s}$	0.02 s
$\beta_{E},\beta_{F},\beta_{v}$	0.4, 0.4, 0.2
$\lambda_{\Omega }$	0.01
$\lambda_{H}$	0.1
$E_{\mathrm{ref}}$	30 J
$v_{\mathrm{ref}}$	1.5 m/s
$R_{\mathrm{th}}$	0.4
$\kappa_{E}$	1.2
$w_{1},w_{2},w_{3},w_{4},w_{5},w_{6}$	1, 100, 10, 0.01, 0.1, 1

**Table 3:** Allowable peak impact-force thresholds for candidate body regions.

Parameter	Value
Chest	1200
Back	1500
Hip/pelvis	1800
Shoulder	900
Elbow	600
Knee	800
Upper arm	700
Forearm	600

## Data Availability

Dataset available on request from the authors.
